# Hormonal and metabolic regulation of tomato fruit sink activity and yield under salinity

**DOI:** 10.1093/jxb/eru347

**Published:** 2014-08-28

**Authors:** Alfonso Albacete, Elena Cantero-Navarro, María E. Balibrea, Dominik K. Großkinsky, María de la Cruz González, Cristina Martínez-Andújar, Ann C. Smigocki, Thomas Roitsch, Francisco Pérez-Alfocea

**Affiliations:** ^1^Department of Plant Nutrition, CEBAS-CSIC, Campus de Espinardo, 30100 Murcia, Spain; ^2^Institute of Plant Sciences, Department of Plant Physiology, University of Graz, 8010 Graz, Austria; ^3^Department of Plant and Environmental Sciences, Copenhagen Plant Science Centre, University of Copenhagen, Højbakkegård Allé 13, DK-2630 Taastrup, Denmark; ^4^Instituto de Bioquímica Vegetal y Fotosíntesis, Universidad de Sevilla, CSIC, 41092 Sevilla, Spain; ^5^Molecular Plant Pathology Laboratory, USDA, ARS, Beltsville, MD 20705, USA; ^6^Global Change Research Centre, Czech Globe AS CR, v.v.i., Drásov 470, Cz-664 24 Drásov, Czech Republic

**Keywords:** Cell wall invertase, cytokinins, fruit, salinity, sink activity, tomato.

## Abstract

Salinization of water and soil has a negative impact on tomato (*Solanum lycopersicum* L.) productivity by reducing growth of sink organs and by inducing senescence in source leaves. It has been hypothesized that yield stability implies the maintenance or increase of sink activity in the reproductive structures, thus contributing to the transport of assimilates from the source leaves through changes in sucrolytic enzymes and their regulation by phytohormones. In this study, classical and functional physiological approaches have been integrated to study the influence of metabolic and hormonal factors on tomato fruit sink activity, growth, and yield: (i) exogenous hormones were applied to plants, and (ii) transgenic plants overexpressing the cell wall invertase (cwInv) gene *CIN1* in the fruits and *de novo* cytokinin (CK) biosynthesis gene *IPT* in the roots were constructed. Although salinity reduces fruit growth, sink activity, and *trans*-zeatin (*t*Z) concentrations, it increases the ethylene precursor 1-aminocyclopropane-1-carboxylic acid (ACC) during the actively growing period (25 days after anthesis). Indeed, exogenous application of the CK analogue kinetin to salinized actively growing fruits recovered sucrolytic activities (mainly cwInv and sucrose synthase), sink strength, and fruit weight, whereas the ethylene-releasing compound ethephon had a negative effect in equivalent non-stressed fruits. Fruit yield was increased by both the constitutive expression of *CIN1* in the fruits (up to 4-fold) or *IPT* in the root (up to 30%), owing to an increase in the fruit number (lower flower abortion) and in fruit weight. This is possibly related to a recovery of sink activity in reproductive tissues due to both (i) increase in sucrolytic activities (cwInv, sucrose synthase, and vacuolar and cytoplasmic invertases) and *t*Z concentration, and (ii) a decrease in the ACC levels and the activity of the invertase inhibitor. This study provides new functional evidences about the role of metabolic and hormonal inter-regulation of local sink processes in controlling tomato fruit sink activity, growth, and yield under salinity.

## Summary

Cytokinins and cell wall invertase are positive players in regulating fruit sink strength, growth, and yield under salinity as components of the same signalling cascade establishing and developing sink organs.

## Introduction

Salinity decreases crop yield by first reducing growth and the number of assimilate-consuming sink organs (both vegetative and reproductive), and then by decreasing assimilate production in photosynthetically active source tissues (Hsiao, 1973; Cramer, 1992; Chazen et al., 1995). Therefore, to sustain crop productivity under unfavourable saline conditions requires the maintenance of assimilate production in source tissues (leaf area and photosynthesis) and also transport to and use within sink and harvestable tissues (Perez-Alfocea et al., 2010; Albacete et al., 2014). Ideally, maintaining or increasing sink activity in the plant (individually or collectively) will help to avoid initial photoinhibition and premature stress-induced senescence, thus maintaining assimilate production and transport in/from the source tissues (Perez-Alfocea et al., 2010; Albacete et al., 2014). For this reason, the discovery of any novel gene involved in stress adaptation should include an investigation of its effect on sink activity and, ultimately, on yield performance. Increasing sink activity in the reproductive organs by either (i) the overexpression of sucrolytic activities, for example under the control of a fruit-/grain-specific promoter, or (ii) the positive (i.e. cytokinins, gibberellins) or negative (i.e. ethylene) modulation of hormonal factors regulating the sink number and activity, could be useful strategies for increasing yield stability under suboptimal abiotic stress conditions (Albacete *et al.*, 2014).

Sink organs of most plant species are supplied with carbon and energy in the form of sucrose which can be used for the biosynthesis of primary metabolites important for tissue growth and development. The growth capacity, evaluated as net accumulation rate of dry matter, is a measure of sink strength (Ho *et al.*, 1989; Marcelis, 1996). Warren-Wilson (1972) proposed that sink strength is the product of multiplying sink size and sink activity. Sink size is a physical factor that includes cell number and cell size, whereas sink activity is a physiological factor that includes multiple components and key enzymes of carbohydrate metabolism and storage, thus maintaining a sucrose gradient and transport between source and sink organs (Ho, 1984). Under salinity, the competition for carbon between different physiological processes and sink organs significantly affects plant growth and crop yield (Munns, 1993; Balibrea *et al.*, 1996, 1999, 2000; Daie, 1996; Perez-Alfocea et al., 2010). The use of sucrose in sink tissues requires cleavage of the glycosidic bond, catalysed by both sucrose synthase (SUS) and invertases. Three types of invertase isoenzymes are distinguished based on their solubility, subcellular localization, pH optima, and isoelectric point: vacuolar invertase (vacInv), cytoplasmic invertase (cytInv), and cell-wall bound or apoplastic invertase (cwInv) (Roitsch and González, 2004). Decreased fruit set and/or tomato fruit weight and, ultimately, crop yield under salinity conditions have been partially explained in terms of sucrose transport and metabolism (Ho, 1996). In this regard, it has been reported that a highly saline treatment (150mM NaCl for 10 d) decreased pollen viability, inducing tomato flower abortion, which was thought to be due to decreased carbohydrate transport from source leaves to the inflorescence and pollen-producing tissues, as suggested by marked reductions in sucrolytic activities of cwInv and SUS (Ghanem *et al.*, 2009). Thus, cwInv seems essential in maintaining sucrose import to sink tissues by regulating the apoplastic sucrose unloading from the phloem (Roitsch and Ehness, 2000; Roitsch et al., 2003; Koch, 2004) during pollen development (Roitsch and González, 2004), especially under potential source-limiting stress conditions (Albacete *et al.*, 2011). Assuming that the flower is fertilized, fruit or grain filling is also a susceptible process limiting yield. For example, tomato yield reduction by low to moderate salinity levels in irrigation water (25–75mM NaCl) is due to decreased fruit weight rather than fruit number (Cuartero and Fernández-Muñoz, 1998), and sucrolytic enzymes have been also implicated. In fact, the inhibition of the cytInv and the use of the accumulated sucrose by other sucrolytic enzymes could be considered as a limiting step and an adaptive response in the control of sucrose import and fruit growth under salinity, respectively (Balibrea *et al.*, 1996, 1999). Although the cytoplasmic cleavage of sucrose has been considered an important regulatory step for assimilate import in tomato fruit (Ho, 1996), cytInv has rarely been studied and most published work only addresses vacInv and SUS. Few studies have shown that cwInv has important functions in establishing and maintaining sink metabolism in tomato fruits and also in phloem unloading, carbohydrate partitioning, and sink growth (Godt and Roitsch, 1997; Sturm, 1999; Roitsch, 1999; Roitsch *et al.*, 2000). An inverse relationship between cytoplasmic sucrolytic activities (CSA) and cwInv in fruits of domestic and hybrid (between *Solanum lycopersicum* and the wild relatives *S. cheesmaniae* and *S. chmielewskii*) tomato plants has been reported as a regulatory mechanism for maintaining sink capacity and dry matter accumulation (Balibrea *et al.*, 2003; Balibrea *et al.*, 2006).

Plant hormones are directly implicated in tomato fruit set and development (reviewed in Ariizumi *et al.* (2013)) and sink-related processes (Ehness and Roitsch, 1997; Roitsch and Ehness, 2000; Roitsch *et al.*, 2003). Exogenous hormonal application to fruits at different developmental stages had highlighted their importance in fruit set and growth (reviewed by De Jong *et al.* (2009)). The effect of 2,4-dichlorophenoxyacetic acid (2,4-D)- and gibberellic acid (GA_3_)-induced fruit set on the expression of diverse genes involved in auxin and gibberellin (GA) signalling has been studied in tomato plants (Serrani et al., 2008; De Jong *et al.*, 2009). Furthermore, increases in indoleacetic acid (IAA), cytokinins (CKs), and bioactive GA concentrations have been found during tomato fruit set and early fruit development, whereas abscisic acid (ABA) decreased (Mariotti *et al.*, 2011). Indeed, endogenous levels of CKs have been linked with fruit growth (Gillaspy *et al.*, 1993; Srivastava and Handa, 2005) and are critically involved in the regulation of early fruit growth through the regulation of cell division by D-type cyclin expression (Baldet *et al.*, 2006). Gene expression profiles have suggested that ethylene is also involved in regulating fruit set (Vriezen *et al.*, 2008). Before tomato fruit development, genes associated with ethylene biosynthesis were strongly expressed and associated with relatively high ethylene concentrations. However, during fruit development, ethylene concentrations decreased owing to an auto-inhibitory effect of *LeACS1A,6* and *LeACO1,3,4* genes (Cara and Giovannoni, 2008).

Information acquired through biochemical, genetic, and molecular studies is now beginning to reveal a possible interlink between hormonal and metabolic factors in the regulation of tomato fruit growth and crop yield under salinity. Elevated levels of CKs have been associated with the upregulation of sink strength and invertase expression (Ehness and Roitsch, 1997). Root CK biosynthesis has been shown to be important in mediating the relationship between decreased shoot CK status and salt-induced changes in growth, senescence, and fruit yield (Albacete *et al.*, 2008; Ghanem et al., 2008; Albacete *et al.*, 2009; Ghanem *et al.*, 2011). Augmenting root-to-shoot CK transport (through grafting CK-overproducing rootstocks) improved vegetative growth and ion homeostasis, delayed leaf senescence, and increased fruit yield of salinized tomato (Albacete *et al.*, 2009; Ghanem *et al.*, 2011). Grafting WT plants onto a constitutively (*35S*) expressing *IPT* rootstock increased fruit yield by 30% compared with salinized self-grafted WT/WT plants. Similarly, reduction of ethylene biosynthesis and perception (reviewed by Stearns and Glick, 2003), mainly focused on the ACC deaminase gene, has been shown to regulate carbon metabolism and sink strength.

Therefore, the aim of this work was to study the influence of both metabolic (sucrose metabolism) and hormonal (auxins, CKs, ABA, GA_3_, and ethylene) factors on fruit sink activity and strength, and hence on overall fruit yield, in tomato plants growing under salinity. In this study, different classical and functional physiological approaches were integrated: the classical exogenous hormonal application and biotechnological overexpression of the cwInv gene *CIN1* in fruits or the CK biosynthesis gene *IPT* in roots.

## Material and methods

### Plant cultivation

Tomato plants (*Solanum lycopersicum* L.) were sown in trays filled with a perlite–vermiculite mixture (1/3, v/v) moistened regularly with half-strength Hoagland’s nutrient solution. Forty days after germination, seedlings were transferred to a polyethylene greenhouse using perlite bags as growing medium. Plants were distributed in a planting pattern of 2 m between rows and 0.5 m between plants within rows, and cultivated with one stem, eliminating all axillary buds. A standard fertilization solution for tomato was applied by a drip irrigation system. Salt treatment started 10 d after the transfer. The fertilization solution was prepared in two 1500-l tanks and the electrical conductivities of the treatments were 1.2 (control) and 8.7 dS m^–1^ (75mM NaCl).

Flowers were tagged at anthesis 30 d after the beginning of the salt treatment, and actively growing fruits were harvested and weighted 25 d after anthesis (DAA). Pericarp tissue was cut in small pieces, frozen with liquid nitrogen, and stored at –80 ºC until analysis. Three replicates were carried out for each fruit harvest and treatment.

### Hormone applications

From an original population of 40 tomato plants (*Solanum lycopersicum* L. cv. Durinta F1, from Western Seed 2000 SL, Almeria, Spain) growing at the greenhouse under a moderate salinity level (75mM NaCl), 25 were selected for exogenous hormonal applications. Two hormonal applications (with an interval of two weeks) were done by spraying directly the fruit trusses at the flowering/early-fruiting stage with four different plant regulators: gibberellic acid (GA_3_), kinetin (KIN), indolacetic acid (IAA) (10^–5^ M) (purchased from Sigma-Aldrich, MO, USA), and ethephon (ET, 0.15% v/v, applied as Ethrel® 48 SL, Bayer CropScience, Monheim am Rhein, Germany). Control plants were sprayed with distilled water.

### Radiolabelled sucrose allocation into developing fruits: sink activity

Forty-day old tomato seedlings (*Solanum lycopersicum* L. cv. Durinta F1) were placed in pots containing peat as substrate. Plants were grown in a growth chamber under 16h daylight period. The air temperature ranged from 25–28 °C during the day and 17–18 °C during the night. Relative humidity was maintained at 70±5% during the night and 50±5% during the day. Light intensity at the top of the canopy was 250 µmol m^–2^ s^–1^. After 5 d of acclimation, seedlings were exposed to 0mM (control) or 100mM NaCl added to the nutrient solution. To study the effects of the exogenous hormonal application on sink activity, KIN (10^–5^ M) and ET (0.15% v/v) were applied to salinized plants and control plants, respectively, 40 d after salt treatment started, by spraying the second fruit truss of each plant. One week later the radioactivity assay was performed. Five plants per treatment were used for this experiment.

The application of the radio-labelled sucrose was made as described by Garrido *et al.* (2002) and Ben Salah *et al.* (2009). When the plants showed 2 trusses of developing fruits (8–12 fruits per plant), 10 µl of a 150 Bq µl^–1^ [^14^C(U)]-sucrose (specific activity 625 mCi mmol^–1^, Nucliber S.A., Madrid, Spain) aqueous solution were applied to a lightly abraded area (about 1cm^2^) in the basal zone of the fruit-feeding mature leaf situated just below the second truss. Ten minutes after application the area was covered with lanoline to avoid desiccation. Plants were kept 24h with solar photoperiod and at room temperature under a cabin of radioactive gas capture. After that, all treated fruits were weighted and cut into small pieces. Total fruit fresh material was separated in 1 g-packages and each one was immersed in a separate vial containing 10ml of scintillation cocktail (OptiPhase “HiSafe” 3, Wallac-PerkinElmer, MA, USA) and stored in darkness at room temperature for 24h. Radioactivity was determined in a Wallac 1400 DSA beta liquid scintillation counter (PerkinElmer, Waltham, MA, USA). Using a standard, the efficiency of the ^14^C counting was calculated as 97.8±1.2%; therefore, the radioactivity measured in the ^14^C-sucrose solution applied to the leaves was 146.7±2.7 Bq µl^–1^. Fruit sink activity and fruit strength were determined as the amount of radioactivity accumulated after that period per gram of FW (Bq g^–1^ FW) or per fruit (Bq fruit^–1^), respectively.

Similar determinations of fruit sink activity and strength were also carried out in two homozygous tomato transgenic lines for the *InvLp6g::CIN1* construct (*CIN1*-91 and *CIN1*-93) and in the wild type (cv. P-73), cultivated under control (0mM NaCl) and saline (100mM NaCl) conditions. Three plants per line were evaluated.

### Overexpressing the cell wall invertase *CIN1* under the control of a fruit-specific promoter

The full-length 1.7kb *CIN1* cDNA under the control of a 2.5kb fragment of the promoter of vacuolar invertase *pInvLp6g* from *Solanum pimpinellifolium* (Elliott *et al.*, 1993) (gene bank accession no. Z12028.1) was cloned into the vector pBI101. After transfer into the *Agrobacterium tumefaciens* strain LBA4404, cotyledons from the semi-indeterminate cv. P-73 of tomato (*Solanum lycopersicum* L.) were transformed with the *CIN1* overexpression construct. T_2_ plants from five different transgenic lines containing the *InvLp6g::CIN1* construct were evaluated in the greenhouse under moderate salinity (75mM NaCl) for fruit yield-related analyses. Plants were identified as homozygous, heterozygous, or azygous for the T-DNA based on the expression of the marker gene *NPTII* that confers resistance to the antibiotics kanamycin and neomycin.

### IPT-transformed cytokinin-overproducing rootstocks

Tomato seeds from different genotypes were sown in a controlled conditions culture chamber. When seedlings had developed three or four true leaves, tomato plants from the cultivar P-73 were grafted (as previously described by Santa-Cruz *et al.* (2002)) onto a determinate commercial cultivar UC-82B (P-73/UC-82B) as well as the same cultivar overexpressing the *IPT* gene (P-73/*IPT*) used as rootstocks. This *IPT* gene codifies for a key enzyme of the CK biosynthesis *isopentenyl transferase* from *Agrobacterium tumefaciens*, and was cloned under the control of the constitutive CaMV *35S* promoter. Seeds were supplied by Dr Ann Smigocki (ARS-USDA, Beltstville, MD, USA; Ghanem *et al.*, 2011). Four plants per combination were transferred to the greenhouse after the grafts had established and a salinity level of 75mM NaCl was applied to the irrigation solution.

### *CIN1* expression analysis

Fresh tissues from leaves, roots, seedlings, and fruits were used for total RNA isolation, and 1 µg of total RNA was used for first strand cDNA synthesis according to standard methods, using oligo(dT) primers. Semi-quantitative RT-PCR using actin to normalize the obtained cDNA amounts was performed as described previously (Großkinsky *et al.*, 2011). For *CIN1* expression analyses the primers CIN1-Forward (5'-CCTGGGAGTATAGTGGCTGAACC-3') and CIN1-Reverse (5'-AGGTCTTCTCTGAATCCG-3') were used.

### Sucrolytic activities and invertase inhibitor enzyme assays

Sucrolytic activities were assayed by determining the NADH delivered in a coupled enzymatic reaction using specific substrates/enzymes depending on the target enzyme (Balibrea *et al.*, 2003; Balibrea *et al.*, 1999). The absorbance was performed at 340nm. Briefly, 0.5g of plant material was homogenized in liquid nitrogen in a mortar and re-suspended in 1ml homogenization buffer (200mM HEPES, 3mM MgCl_2_, 1mM EDTA, 2% glycerol, 0.1mM PMSF, 1mM benzamidine). The homogenate was mixed for 20min at 4 °C and centrifuged for 15min at 10 500 *g* and 4 °C. The supernatant was used for soluble enzyme preparation. The pellet (cell-wall fraction) was washed three times with distilled water and re-suspended in 200mM HEPES, 3mM MgCl_2_, 15mM EDTA, 2% glycerol, 0.1mM PMSF, 1mM benzamidine, 1M NaCl. CwInv and vacInv activities were measured at pH 5, and cytInv activity at pH 7. SUS activity was assayed in the same reaction medium than cytInv, but with the addition of UDP. The proteins were analysed with Bradford reagent using BSA as standard. The invertase inhibitor assay was performed as previously described (Bonfig *et al.*, 2010).

### Sugar determination

A total of 100mg plant material was ground in liquid nitrogen and 0.9ml water was added. After homogenization with cationic and anionic exchange resins and centrifugation for 10min at 20 000 *g* and 4 °C, the supernatant was filtered and 10 µl were injected in a normal-phase liquid chromatography system (Shimadzu Corporation, Kyoto, Japan), using acetonitrile/water (85/15, v/v) as the mobile phase at a flow rate of 1ml min^–1^ (Balibrea *et al.*, 2000, 2003).

### Hormone extraction and analysis

Hormones were analysed as described previously (Albacete *et al.*, 2008). Briefly, 0.5g of each sample were homogenized, in triplicate, with liquid nitrogen, and extracted with 2.5ml of methanol:H_2_O (80:20). Samples were centrifuged and the precipitates were re-extracted with another 2.5ml of the methanol:H_2_O mixture. The two supernatants were mixed and passed through a SepPak Plus C18 cartridge (SepPak Plus, Waters, Millford, MA, USA). Samples were evaporated and the residues were dissolved in a methanol:H_2_O (20:80) mixture and filtrated. 8 µl of sample were injected in an Agilent 1100 Series HPLC (AgilentTechnologies, Santa Clara, CA, USA), equipped with a micro-wellplate autosampler and a capillary pump, connected to an Agilent Ion Trap XCT Plus mass spectrometer (Agilent Technologies, Santa Clara, CA,USA) using an electrospray interface.

### Statistics

All experiments were repeated three times, and results of one representative experiment are presented in each case. Data were subjected to an analysis of variance (ANOVA) using the SPSS software (Version 19.0, SPSS Inc., Chicago, IL, USA). The statistical significance of the results was analysed by Student-Newman-Keuls test at the 5% level.

## Results

### Salt effect on [^14^C(U)]-sucrose allocation into developing fruits: a measurement of sink activity

**Fig. 1.**
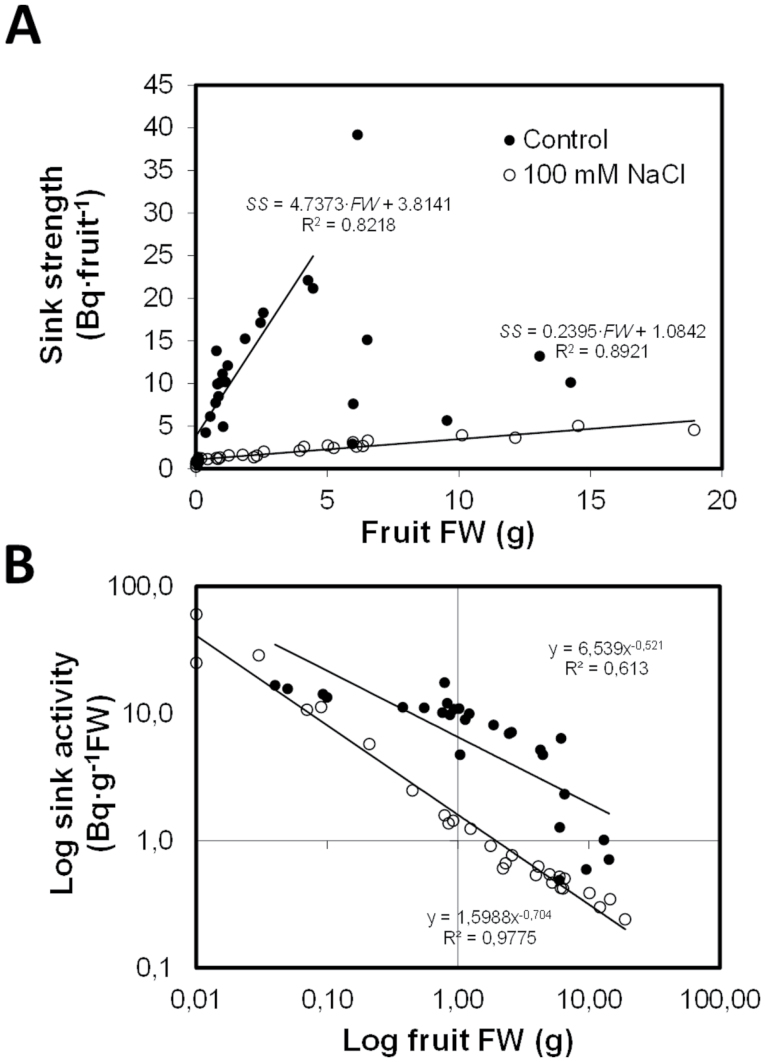
Linear correlations between fruit sink strength (A, measured as the amount of radioactivity accumulated in Bq) or fruit sink activity (B, expressed as logarithm of the amount of radioactivity accumulated in Bq per gram of fresh weight), and the fruit fresh weight of tomato plants (cv. Durinta F1) cultivated for 40 d in the absence (closed circles) or presence (open circles) of 100mM NaCl.

The capacity of developing fruits to attract [^14^C(U)]-sucrose from a source leaf, measured as the amount of radioactivity (Bq) accumulated per gram of fresh weight (Bq g^–1^ FW), has been considered as an estimation of the sink activity of the fruits. The result of multiplying sink activity by the sink size was considered as the fruit sink strength, expressed in Bq fruit^–1^. As a consequence, sink activity can be regarded as a metabolic parameter strongly determining the fruit sink strength and the capacity of the fruit to attract assimilates from the active fruit-feeding photosynthetic tissues. Results clearly show that 100mM NaCl applied to the irrigation solution during 40 d provoked a 3-fold decrease in both sink strength (Fig. 1A) and sink activity (Fig. 1B) in developing fruits up to 5–7g FW as compared with the control. Both parameters were also lower in the salinized fruits weighing more than 7g but the reduction was difficult to quantify owing to the high dispersion of data in the control fruit. This dispersion may be explained by the interaction between different fruit sizes, truss position, and fruit position on a truss and the limited availability of the labelled sucrose.

### Effects of exogenous hormonal application

#### Fruit growth

**Fig. 2.**
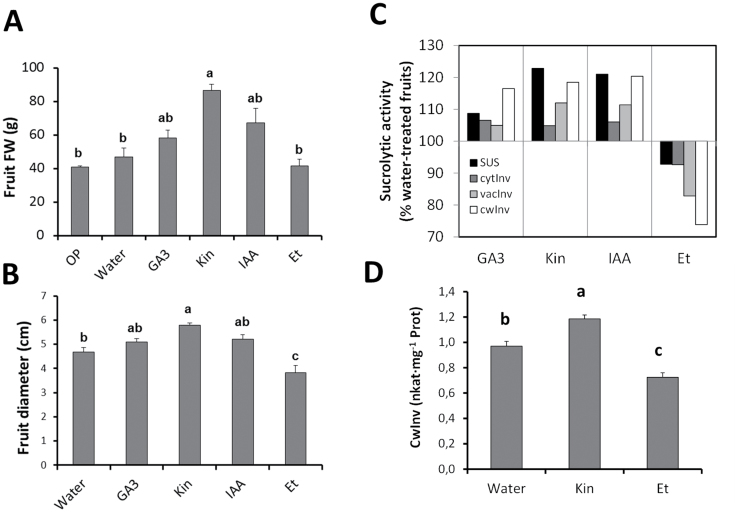
Final fruit fresh weight (A) and equatorial diameter (B) of tomato fruit (cv. Durinta F1) sprayed with different hormones and cultivated in the presence of 75mM NaCl. Sucrolytic activities in percentage relate to the water-treated fruits (C) and cell wall invertase activity (D) of actively growing tomato fruits (25 DAA) sprayed with different hormones and cultivated in the presence of 75mM NaCl. Data are means of 6 plants±SE. Values marked with a same letter are not significantly different at *P*<0.05 according to the Student–Newman–Keuls test. OP = original population.

The application of 10^–5^ M of GA_3_, KIN, or IAA to a truss of actively growing fruits on salinized plants increased the final fresh weight of mature fruits by 1.3-, 2-, and 1.6-fold, respectively, as compared with the average of the whole population and water-sprayed fruits (Fig. 2A). The ethylene-releasing chemical Ethephon (ET) had no effect on the final fruit weight but it significantly reduced the equatorial diameter of the fruit (Fig. 2B).

#### Sucrolytic activities

The activity of sucrose-cleaving enzymes significantly increased in the salinized fruit after treatment with GA_3_, KIN, or IAA, whereas the ET treatment inhibited the enzymes when compared with the water-sprayed fruits (Fig. 2C). The activity of the cwInv enzyme was up to 1.2-fold higher in GA_3_-, KIN-, and IAA-treated plants, but the activity was inhibited 28% by the ET treatment. KIN and IAA induced SUS activity 1.2-fold and GA_3_ 1.1-fold. Similar differences between treatments were observed for vacInv, but to a lower extent (5–15%). CytInv activity was the least induced by the three hormonal treatments (5–8%). In addition to cwInv, *Et al*so inhibited the cytoplasmic sucrolytic activities of SUS and cytInv (8%), and the vacInv (15%) compared with the water-sprayed fruits (Fig. 2C). Regarding the absolute values of cwInv, exogenous applications of KIN (10^–5^ M) and ET (0.15%) to actively growing salinized fruits provoked a significant increase (KIN) or decrease (ET) of this sucrolytic enzyme compared with the water-treated fruits (Fig. 2D), suggesting an important role for cwInv in the regulation of hormonal-mediated sink activity in tomato fruits growing under salinity. Those hormones were selected for further experiments as positive and negative regulators of fruit sink activity.

### Cytokinin (kinetin) and ethylene (ethephon) effects on sink activity

**Fig. 3.**
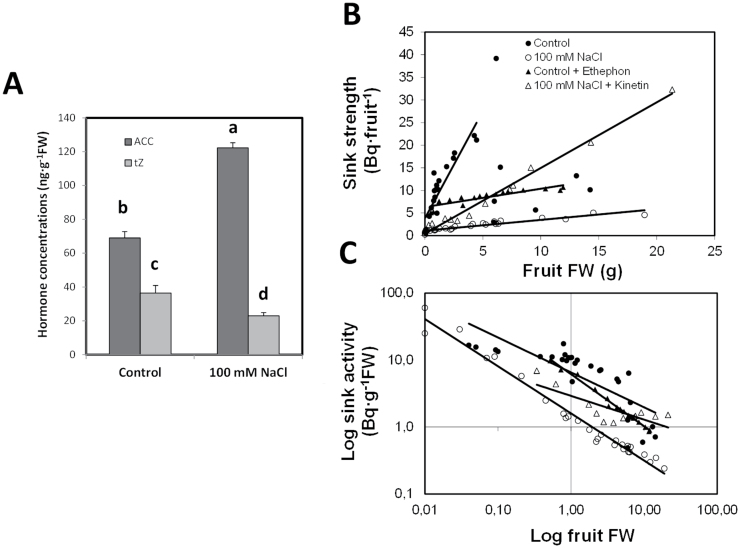
*Trans*-Zeatin (*t*Z) and 1-aminocyclopropane-1-carboxylic acid (ACC) concentrations (A) in actively growing fruits (25 DDA) of tomato plants cultivated for 40 d in the absence (light-grey bars) or presence (dark-grey bars) of 100mM NaCl. Data are means of 6 plants±SE. Values marked with a same letter are not significantly different at *P*<0.05 according to the Student-Newman-Keuls test. Linear correlations between fruit sink strength (B, measured as the amount of radioactivity accumulated in Bq) or fruit sink activity (C, expressed as logarithm of the amount of radioactivity accumulated in Bq per gram of fresh weight), and the fruit fresh weight of non-salinized (sprayed with ethephon) and salinized (100mM NaCl, treated with kinetin) tomato fruits (cv. Durinta F1).

Analyses of the endogenous levels of the most bioactive CK in tomato, *t*Z, and the ethylene precursor ACC showed an important reduction of the *t*Z concentrations in salinized developing (green stage, 25 d after anthesis; DAA) fruits (50%), whereas ACC concentrations significantly increased (2-fold) (Fig. 3A). To test if hormonal factors have an effect on fruit sink activity that was measured as sucrose import, KIN, the best positive effector, and ET, the negative effector, were applied to salt-stressed plants. Application of KIN recovered sink activity (Bq g^–1^ FW), and thereafter sink strength, in salinized fruits reaching similar slope values (as a function of fruit size) as compared with the non-stressed control fruits (Fig. 3B, C). A similar but negative effect was observed when the ethylene-releasing chemical ET was applied to control fruits, attaining in this case similar slope values as the salinized fruits (Fig. 3B, C). Therefore, the exogenous application of CKs mimics the absence of stress in salinized fruits, whereas the ethylene mimics the salt effect in control fruits, thus supporting the suggestion that CK and ethylene are endogenous effectors regulating sink activity in tomato fruit grown under salinity.

### Effect of the cell wall invertase gene *CIN1* overexpression under the control of a putative fruit-specific promoter

#### Fruit-yield related parameters

**Fig. 4.**
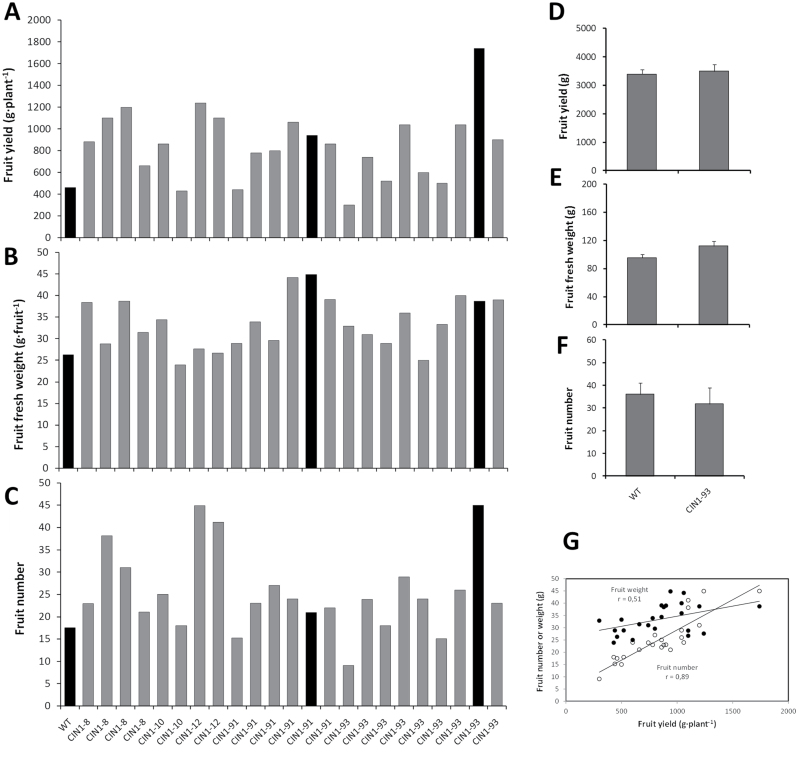
Fruit yield (A), fruit fresh weight (B), and number of fruits (C) of wild-type tomato plants (cv. P-73) and a segregating T_2_ tomato population containing the *InvLp6g::CIN1* construct and cultivated under moderate salinity (75mM NaCl). Fruit yield (D), fruit fresh weight (E), and number of fruits (F) of wild-type tomato plants (cv. P-73) and the selected CIN1-93 line the*InvLp6g::CIN1* construct and cultivated under control conditions. Linear correlations (G) between fruit number (open circles) or weight (closed circles) and fruit yield. Black bars indicate selected lines. Data are presented as means±SE.

**Fig. 5.**
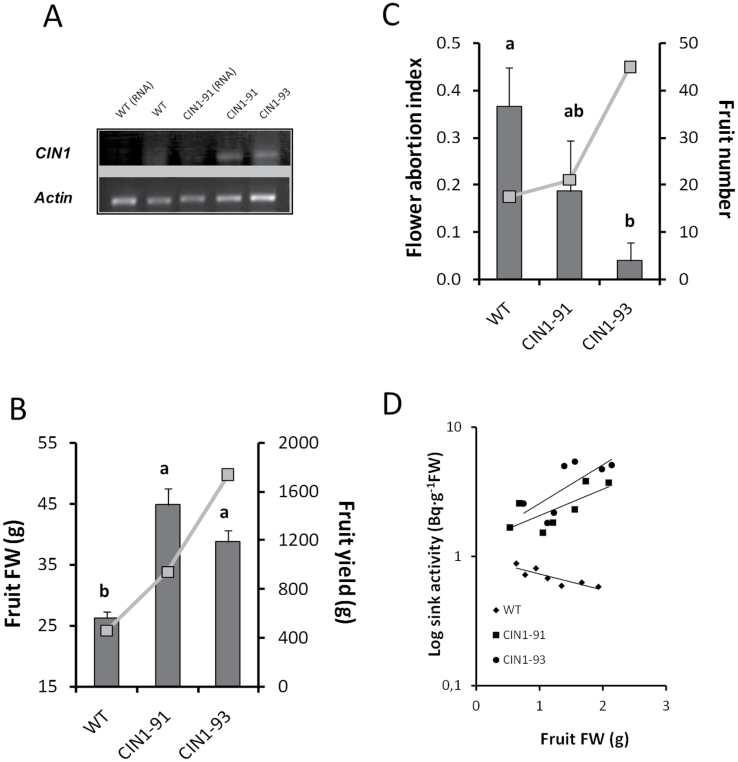
*CIN1* expression in the fruit (A), fruit fresh weight (bars) and fruit yield (line) (B), flower abortion index (bars) and fruit number (line) (C), and linear correlations between fruit sink activity (logarithmic scale) and fruit fresh weight (D) of wild-type tomato plants (cv. P-73) and two selected homozygous lines expressing the *InvLp6g::CIN1* construct and cultivated under moderate salinity (75mM NaCl). Data are means of 3 plants±SE. Values marked with a same letter are not significantly different at *P*<0.05 according to the Student–Newman–Keuls test.

To test the hypothesis that the specific increase of invertase activity in the fruit could recover sink strength and fruit growth under suboptimal conditions imposed by salinity, we generated transgenic tomato plants overexpressing the cwInv gene *CIN1* from *Chenopodium rubrum* under the control of the putative fruit-specific promoter from the vacuolar invertase gene *InvLp6g* of *Solanum pimpinellifolium* (Klann *et al.*, 1993). *Solanum lycopersicum* cv. P-73 plants were transformed with the *pInvLp6g::CIN1* construct and a total of 23 T_2_ segregating plants were analysed under moderate salinity (75mM NaCl) for parameters related to fruit yield. Of these, 9 plants generated homozygous and 11 plants heterozygous T_3_ progenies based on kanamycin resistance, whereas 3 plants produced 100% kanamycin-sensitive progenies. Fruit yield was strongly increased for several lines (Fig. 4A) evident by higher fruit fresh weight (Fig. 4B) and/or higher fruit number (Fig. 4C), whereas yield-related parameters under control conditions were not affected (Fig. 4D–F). Fruit yield under moderate salinity correlated stronger with fruit number (*r* = 0.89, *P*≤0.01, *n* = 26) than individual fruit fresh weight (*r* = 0.51, *P*≤0.01, *n* = 26) (Fig. 4G). Based on these results, salt stress tolerance during fruit development was investigated for two selected homozygous *CIN1* lines, which showed strong *CIN1* expression (Fig. 5A), one with increased fruit number (*CIN1*-93) and other one with higher fruit weight (*CIN1*-91), to distinguish between a more likely systemic effect on the fertilization and fruit set and a more sink-specific effect on fruit growth. Fruit yield (Fig. 5B, line), fruit fresh weight (Fig. 5B, bars), and fruit number (Fig. 5C, line) were higher, whereas flower abortion index was reduced (Fig. 5C, bars) in both *CIN1* lines under salt stress. Importantly, fruits from *CIN1* plants exhibited from 5- up to 15-fold increase in sink activity, measured by the capacity to attract [^14^C(U)]-sucrose from a truss-feeding mature leaf with respect to WT fruits (Fig. 5D). This shows a direct functional correlation between higher cwInv *CIN1* gene expression (and activity) and increased sink activity (and strength) in salinized fruits.

#### Sugar concentrations, sucrolytic activities, and invertase inhibitor in the fruits

**Fig. 6.**
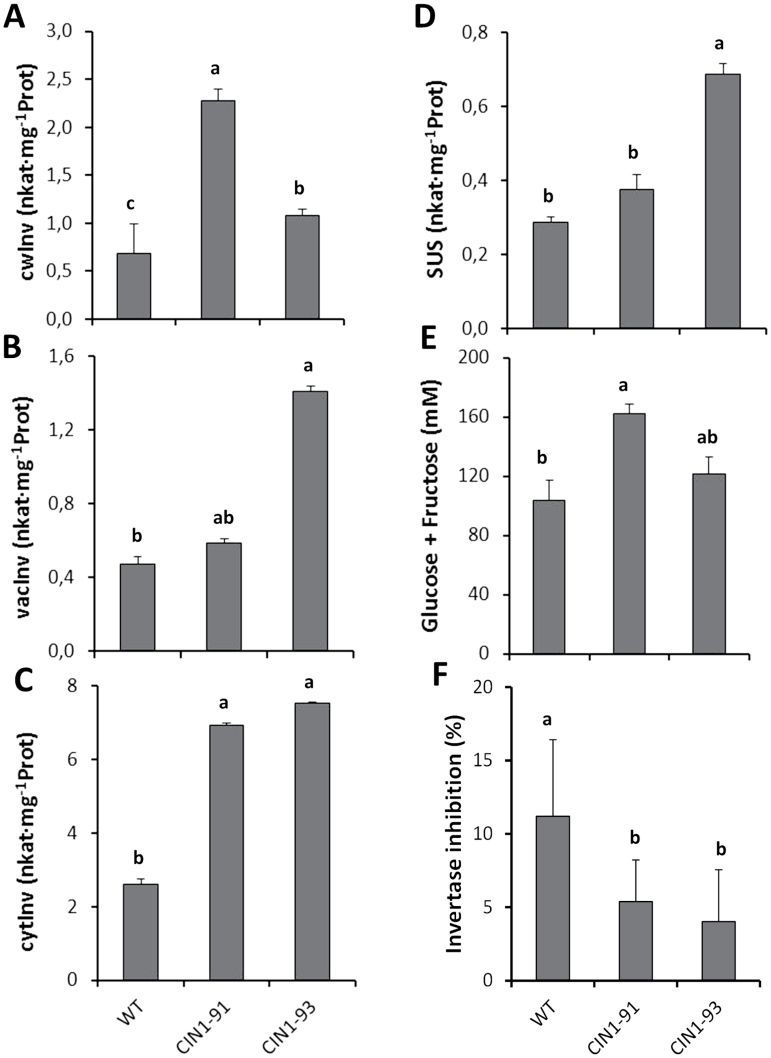
Cell wall invertase (A), vacuolar invertase (B), cytoplasmic invertase (C), and sucrose synthase (D) activities, hexose (glucose + fructose) concentrations (E), and invertase inhibitor activity (F) in tomato fruits of wild-type tomato plants (cv. P-73) and two selected homozygous lines expressing the *InvLp6g::CIN1* construct and cultivated under moderate salinity (75mM NaCl). Data are means of 3 plants±SE. Values marked with a same letter are not significantly different at *P*<0.05 according to the Student–Newman–Keuls test.

A significantly higher cwInv activity was observed in actively growing fruits (25 DDA) of the selected homozygous lines, from 1.6-fold in line *CIN1*-93 up to 3-fold in line *CIN1*-91 (Fig. 6A), accompanied by an increase (up to 3-fold) in the other sucrolytic activities: vacInv, sucrose synthase, and especially cytInv (Fig. 6B–D). The increased sucrolytic activity resulted in a 1.6- and 1.2-fold increase in hexose concentration in fruits of *CIN1-91* and *CIN1-93* lines, respectively (Fig. 6E). Most studies on sucrose metabolism under abiotic stress have focused only on the transcriptional regulation of invertases and the invertase activity present in extracts. Therefore, we have addressed the possible role of invertase inhibitors in post-translational regulation of invertase activity. Invertase inhibitor activity was significantly lower (2-fold) in *CIN1* fruits (Fig. 6F), thus further contributing to the maintenance of sink strength under saline conditions.

#### Fruit hormonal concentrations

**Fig. 7.**
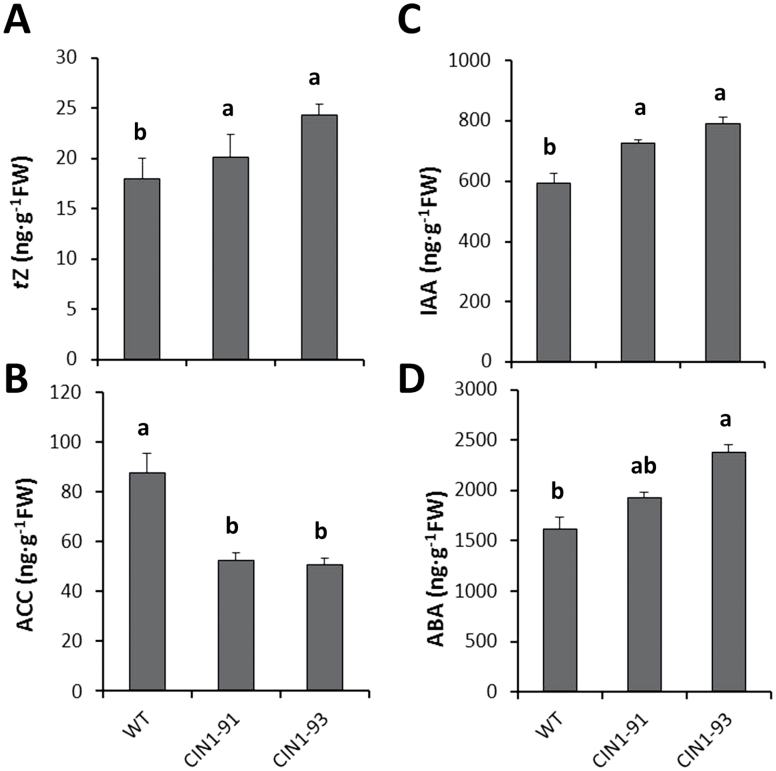
*Trans*-Zeatin (A), 1-aminocyclopropane-1-carboxylic (B), indoleacetic acid (C), and abscisic acid (D) concentrations in tomato fruits of wild-type tomato plants (cv. P-73) and two selected homozygous lines expressing the *InvLp6g::CIN1* construct and cultivated under moderate salinity (75mM NaCl). Data are means of 3 plants±SE. Values marked with a same letter are not significantly different at *P*<0.05 according to the Student–Newman–Keuls test.

To differentiate between the metabolic effect of the transgene on the fruit sink activity from other putative hormonal effects mediated by the transformation events, the concentrations of the active CK *t*Z, the auxin IAA, the ABA, and the ethylene precursor ACC were determined in actively growing fruits of the selected homozygous lines and WT plants. Concentrations of *t*Z significantly increased up to 35% in the fruits of the selected lines with respect to WT (Fig. 7A). In contrast, the ethylene precursor ACC was 1.5 times higher in WT fruits than in *CIN1*-91 and *CIN1*-93 plants (Fig. 7B). Auxin and ABA levels were also increased by 20–30% in the transgenic *CIN1* fruits, compared with the WT (Fig. 7C, D).

### Effect of IPT-transformed rootstocks on scion fruit yield, sucrose metabolism, and hormonal concentration

**Fig 8.**
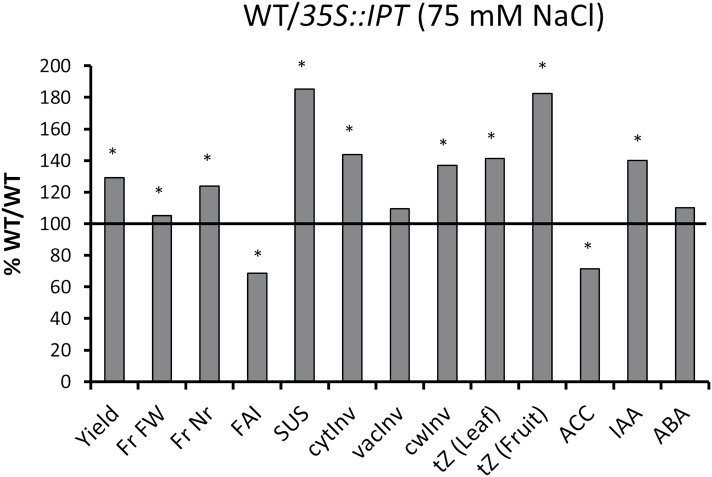
Yield-related (re-plotted from Ghanem *et al.* 2011), hormonal, and metabolic parameters in salinized (75mM NaCl) tomato plants (cv. P-73) grafted onto rootstocks overexpressing the *35::IPT* construct (WT/*IPT*), denoted in percentage with respect to grafted tomato plants (cv. P-73) onto UC-82B rootstocks (WT/WT). Asterisks indicate statistical differences at *P*<0.05 according to the Student–Newman–Keuls test.

To test if the fruit growth under salinity could be enhanced by increasing the endogenous supply of CKs from the roots, plants from the cultivar P-73 were grafted onto transgenic UC-82B rootstocks that overexpress the key enzyme for *de novo* CK biosynthesis (isopentenyl transferase, IPT) from *Agrobacterium tumefaciens* (WT/*IPT*) and compared with grafted plants from the same cultivar P-73 grafted onto UC-82B wild-type plants (WT/WT) (Fig. 8). Fruit yield increased by 30% in WT/*IPT* grafted plants cultivated under moderate salinity (75mM NaCl). The fruit yield increase was primarily attributed to a 25% increase in fruit number (lower flower abortion) and to a significant 5% increase in individual fruit weight (Fig. 8, re-plotted from Ghanem *et al.*, 2011).

The improvement in fruit yield in the chimeric P-73/UC-82B grafted plants was correlated to an increase in *t*Z concentration in mature leaves (40%) and developing (20–35 DAA) fruits (80%) (Fig. 8, re-plotted from Ghanem *et al.*, 2011). Additionally, the fruits registered a 40% increase in IAA and a 30% decrease in ACC concentrations as compared with WT/WT plants. A concomitant increase in sucrolytic activities was also observed in the developing fruits. CwInv and cytInv activities increased by 40% and SUS by 80% (Fig. 8), registering a similar response to root-sourced CKs as exogenous KIN application (Fig. 2C).

## Discussion

### Salinity decreases tomato fruit yield by reducing fruit sink activity

Under salinity stress with low assimilate availability, fruit yield is affected by a reduction in fruit set as well as cell division in early fruit development, a main limiting factor for fruit growth, and cell enlargement during the latter stages of fruit development (Bertin *et al.*, 2001). Tomato yield reduction by low to moderate salinity levels (25–75mM NaCl, 3–8 dSm^–1^) is primarily due to decreased fruit weight, whereas at high salinity (>75–100mM NaCl, >8–10 dSm^–1^) levels, it is the decreased number of fruit that is responsible for reduced yields (Cuartero and Fernández-Muñoz, 1998). Therefore, processes involved in fruit set and development must be responsible for yield reduction under salinity. Decreased pollen viability and increased flower abortion under high salinity (150mM NaCl for 10 d) was explained by decreased carbohydrate availability in the inflorescence and pollen-producing tissues (Ghanem *et al.*, 2009), in spite of increased carbohydrate concentrations within source leaves. This is probably due to reduced transport from source leaves and decreased sink activity (strong reductions in cwInv and SUS activities) during floral development and maturation. Among sucrolytic activities, cwInv seems essential in maintaining sucrose import to sink tissues (Roitsch *et al.*, 2000, 2003) during pollen development (Roitsch and González, 2004). Although a low assimilate supply to the inflorescence is a major cause of flower abortion, a growing fruit has the priority for assimilates compared with flowers (Ho, 1996). Therefore, assuming floral fertilisation is not limited, tomato yield is mainly dependent upon assimilate import and accumulation into individual fruits. In general, salinity decreased both sink strength and sink activity measured as absolute and relative rates of dry matter accumulation during early fruit development (20 d after anthesis until start of ripening) and as assimilate (sucrose) import (Fig. 1). Decreased sink activity was related to sucrose accumulation (up to 30-fold higher than in the control), and to decreased activity of the apoplastic and cytoplasmic sucrose cleaving enzymes; namely, cwInv, cytInv, and SUS (Balibrea *et al.*, 1996, 1999, 2003).

Yin *et al.* (2010) reported that tomato fruit (cv. Micro-Tom) growing under high salinity (160mM NaCl) showed a reduced (30–50%) ^13^C accumulation in developing fruits (10–26 DAA) after 24h of exposure to ^13^CO_2_ feeding, but after 48h, the ^13^C accumulation was 4–8 times that of control fruit. This accumulation was explained by an increased assimilate transport from source leaves to the fruit (supported by the increased expression of the sucrose transporter *LeSUT1*) and increased sucrose-induced starch metabolism (AGPase induction) and accumulation (Yin *et al.*, 2010). During this early growing period under salinity, sucrose and starch accumulated, whereas hexose concentration decreased in normal cultivated tomato (Balibrea *et al.*, 1999, 2003) and the dwarf cv. Micro-Tom (Yin *et al.*, 2010). Although initially this carbohydrate accumulation can be regarded as an increase in fruit sink strength, it could be a consequence of some impairment in sucrose metabolism because of the reduced activity of sucrolytic enzymes (namely the cytoplasmic SUS and cytInv that regulate symplastic sucrose unloading in the phloem). The starch-driven sink strength can be quickly saturated, whereas more hexoses are required for growth and osmotic adjustment in the vacuole. Because of the increased viscosity in the phloem and the changes in assimilate partitioning to other organs under stress (i.e. roots), the reduced fruit weight under salinity can be due to insufficient sink strength owing to a decrease in either absolute or relative sink activity with respect to the reduced and/or more competitive demand for available photoassimilates. Radiolabelled-sucrose assays revealed that salinity strongly reduced the capacity of the fruits to attract assimilates (Fig. 1), which can be explained by a reduced absolute or relative sink activity during early fruit development owing to altered sucrose metabolism (Balibrea *et al.*, 1999; Yin *et al.*, 2010). These changes in sink activity could be mediated by changes in hormone concentrations (Hartig and Beck, 2006; Pérez-Alfocea *et al.*, 2010; Van der Werf and Nagel, 1996).

### Cytokinins and ethylene regulate fruit sink activity

Based on (i) the effects of the exogenous KIN and ethylene-releasing compound ET on both fruit growth under salinity and alteration of sink activity (sucrose import) under control or saline conditions (Figs 2A–D; 3B, C); on (ii) the endogenous changes of the active CK *t*Z and the ethylene-precursor ACC in leaves (Albacete *et al.*, 2008; Ghanem *et al.*, 2008; Ghanem *et al.*, 2011) and fruits (Fig. 3A; Ghanem *et al.* (2011)); and on (iii) the endogenous increases in *t*Z, sucrolytic enzyme activities, and growth in fruits from grafted WT/*IPT* plants (Fig. 8), it can be stated that both hormonal factors (CKs and ethylene) exert a regulatory role on the sink activity of the fruits growing under saline conditions. It is well known that plant growth is modulated by the sink strength, and CKs may regulate rate-limiting steps that determine the availability of nutrients. Indicative for this capacity is the ability of CKs to establish local metabolic sinks, which has been initially demonstrated by the mobilization of radiolabelled nutrients such as amino acids or sugars from other parts of the plant to CK-treated areas (Mothes et al., 1961; Kuiper, 1993) or with local expression of the CK-biosynthetic *IPT* gene (Guivarc’h *et al.*, 2002). Indeed, the level of *t*Z and CK biosynthetic genes is up-regulated at five DAA in tomato, with a positive correlation between *t*Z and cell division. Moreover, the application of synthetic CK to pre-anthesis ovaries resulted in parthenocarpic fruit formation by activating cell division (phase II) (Matsuo *et al.*, 2012). Thus, CK acts as a positive regulator of fruit growth and may also induce parthenocarpy that results in smaller fruits than the pollinated ones (Matsuo *et al.*, 2012; Ariizumi *et al.*, 2013). It has been proposed that CK may be a root-derived signal that controls uptake and utilization of assimilates and biomass distribution, with capacity to change sinks priorities (Beck, 1996; Sakakibara, 2006). Sucrose transported into the sink tissue can be cleaved by sucrose synthases or invertases, the activities of the latter being more dominant during sink initiation and expansion growth (reviewed by Koch (2004)).

The application of KIN to growing fruits or the increase of endogenous CK *t*Z when plants are grown under salinity partially restored sink activity and fruit growth, and also induced the activity of most sucrolytic cleaving enzymes in those fruits (Figs 2C, D and 8). Although no clear relationship can be established between growth recovery by endogenous or exogenous CKs and the induction of any specific sucrolytic activity, cwInv seems to have a predominant role in the regulation of sucrose unloading pathway in tomato fruits (Eschrich, 1980; Damon *et al.*, 1988; Ruan and Patrick, 1995; Ho, 1996; McCurdy *et al.*, 2010) affecting both hexose and biomass accumulation (Balibrea *et al.*, 1999; McCurdy *et al.*, 2010). Indeed, CKs, GAs, brassinosteroids, and auxins have been found to regulate the expression of the cwInv gene *CIN1* positively (Mayak and Borochov, 1984; Ehness and Roitsch, 1997; Roitsch *et al.*, 2000; Roitsch *et al.*, 2003), whereas ethylene was shown to be a negative regulator (Linden *et al.*, 1996). The same regulatory pattern has been found for other sucrolytic enzymes (Roitsch and González, 2004) in different model systems, thus linking hormones, sucrose metabolism, and sink activity. The contrasting effect of the ethylene-related compound ET was shown to reduce the equatorial diameter of salinized fruits but not their fresh weight (Fig. 2A, B), although all the sucrolytic activities were depressed (Fig. 2C). These results suggest that the additional ethylene provision under salinity could only have a subsequent negative effect on cell elongation but not on cell filling. Cell filling seems to be related to the vacuolar capacity of accumulating solutes, which, in turn, could be associated to vacInv, the enzyme activity less affected by ethylene (Fig. 2C).

Therefore, increases in fruit CK concentration and/or cwInv activity could be valid strategies to increase fruit sink strength, growth, and yield under salinity as components of the same signalling cascade establishing and developing sink organs.

### Ectopic increase of extracellular invertase activity and cytokinin levels recover fruit sink activity and crop yield under salinity

As could be expected from the working hypothesis, the specific induction of a sucrolytic enzyme and/or increasing the CK concentration in the fruit could recover the sink activity and fruit growth under the suboptimal limiting conditions imposed by salinity. Under these conditions, not only photosynthesis is reduced, but also the limited carbohydrates available have to be partitioned between interrelated and competitive processes of growth (Balibrea et al., 2000; Munns, 2002; Albacete et al., 2008; Munns and Tester, 2008), osmotic adjustment, and defence against the stress. This recovery in fruit sink activity means a lower inhibition under salinity or even a relative increase with respect to other competitive organs such as the root (Albacete *et al.*, 2008).

Fruit yield was similar in all genotypes under control conditions, but it was more affected by salinity in the wild types: 85% and 72% yield reduction in WT and WT/WT plants, respectively, compared with 55% and 60% in *CIN1* and WT/*IPT* plants, respectively (Figs 4A, D, and 8; Table 2 in Ghanem *et al.* 2011). The increased fruit yield in salinized *CIN1* and WT/*IPT* plants was due to both increases in fruit number and fruit weight and can be explained by local physiological processes in reproductive structures, resulting in a reduced flower abortion index and increased heterotrophic fruit sink metabolism and growth. Up-regulation of extracellular invertase activity by introgression of *LeLIN5* (Baxter *et al.*, 2005), or its down-regulation by RNAi-mediated suppression (Zanor *et al.*, 2009), already demonstrated that extracellular invertases play a key role in inﬂuencing sugar ﬂuxes into, and within developing tomato fruit. *LIN5*, and possibly other extracellular invertases, are expressed in fruit vascular bundles (Fridman *et al.*, 2004) and, in particular, vascular parenchyma cells (Jin *et al.*, 2009), and their activities generate steep sucrose concentration differences from phloem symplasm to apoplasm. However, although extracellular invertase activity has been assumed to be a key determinant of sink activity, this has only now been functionally proven in the *CIN1* transgenic plants (Fig. 5D), as interplay of elevated cwInv activity (Fig. 6A) and *t*Z levels (Fig. 7A), and reduced invertase inhibitor activity (Fig. 6F) and ACC concentration (Fig. 7B).

The elevated levels of CKs in the *CIN1* fruits (Fig. 7A) are also consistent with a function both in the up-regulation of sink strength and invertase expression (Ehness and Roitsch, 1997), whereas the causal relationship cannot be resolved and feedback regulatory mechanisms have to be assumed. A higher cwInv activity could induce cell division through increased CK levels in *CIN1* plants and also by adjusting sink strength to carbohydrate availability. In respect to the decrease in ACC concentration, it has been suggested that invertases have an important function in the enhancement of assimilate import into growing sink tissues (Roitsch *et al.*, 2003; Roitsch and González, 2004), which could indirectly also lead to the repression of ethylene biosynthesis (Mayak and Borochov, 1984). Although induced expression of ethylene biosynthetic genes and ethylene concentrations have suggested that this hormone is involved in regulating fruit set before fruit development, a subsequent drop in ethylene concentration has been observed during the growing stage period (Vriezen *et al.*, 2008). Therefore, as salinity induces ACC accumulation (Ghanem *et al.* 2008; Fig. 3A), the reduced ACC levels in the *CIN1* fruits (Fig. 7B) could alleviate the negative impact of ethylene on invertase expression (Linden *et al.*, 1996) and activity (Fig. 2C, D), thus further contributing to a higher fruit sink activity and strength (Figs 3B, C; 5D). The interplay between *CIN1* expression and levels of IAA and ABA (Fig. 7C, D) in developing fruits needs further investigation. However, increased levels of these hormones could positively influence both fruit set during phase I (IAA) and cell expansion during phase III (IAA and ABA), thus complementing the effect of CKs on both phases I and II (Matsuo et al., 2012; Ariizumi et al., 2013). Similarly, the interplay between increased *t*Z and IAA and decreased ACC in developing fruits from WT/*IPT* plants (Fig. 8) must be investigated, and a possible interaction with sucrose metabolism cannot be ruled out, as suggested by similar results in *CIN1* fruits (Fig. 7).

Therefore, various localized physiological changes conferred by the expression of the *CIN1* gene in the tomato fruit seem to be connected both with hormonal (CKs and ethylene) and sugar metabolism, which resulted in a considerable increase in fruit yield under salinity (Figs 4A–C; 5B, C). In addition to local effects, a systemic effect in the source leaves cannot be ruled out as *CIN1* cwInv is also an essential component of the delay of natural and dark-induced senescence by CKs (Lara *et al.*, 2004). Moreover, it has been reported that silencing the cwInv inhibitor delayed ABA-induced leaf senescence, and increased seed weight and fruit hexose contents in tomato (Jin *et al.*, 2009). A putative expression of *CIN1* in the leaves (data not shown) could delay salt-induced senescence, improving source strength and assimilate supply to flowers, thus decreasing flower abortion under stress (Figs. 4C; 5C). The inverse relationship between number of fruits and fruit weight (Figs 4B, C; 5B, C) in different transgenic lines under moderate salinity suggest a competitive effect between fertilized flowers and growing fruits for the still limiting assimilates (Bohner and Bangerth, 1988; Ho, 1996; Bertin et al., 2001; Baldet et al., 2006). Indeed, the absence of any effect of *CIN1* overexpression on fruit yield parameters under control conditions (Fig. 4D–F) is supported by the fact that carbon assimilation in tomato plants is genetically sink- rather than source-limited (Hocking and Steer, 1994; Albacete *et al.*, 2008; Albacete *et al.*, 2009). Similar absence of any yield-related effect under optimal conditions has been reported in CK-overproducing plants either grafted onto constitutive (*35S*) (Ghanem *et al.*, 2011) or senescence-autoregulated (*SAG12, SAG13*) (Swartzberg *et al.*, 2006) *IPT*-overexpressing plants.

Further analyses of the *CIN1* overexpressing plants revealed that the transgene was surprisingly not only expressed in the fruits, as expected from the published specificity of the promoter used, but also in vegetative parts of the plants. This ectopic expression caused also systemic effects markedly improving water stress adaptation through an efficient physiological strategy of drought avoidance (Albacete *et al*. unpublished results).

## Conclusion

As (i) the fruit yield and the fruit number were similar in all genotypes under control conditions but more affected by salinity in the wild types (P-73 and P-73/UC-82B), and (ii) the fruit weight and number under salinity increased in both transgenic (*CIN1* and P-73/*IPT*) plants as compared with the controls, it can be concluded that both the hormonal *t*Z (cytokinin) and the metabolic *CIN1* (cwInv) factors increase fruit yield under salinity not only by enhancing the individual fruit weight but also by reducing flower abortion and maintaining the number of fruit closer to the non-stressed conditions. These results suggest a direct systemic effect on the overall plant status (as could be expected from the hormonal effector) and/or an indirect effect on the plant status of a locally (fruit) induced process (sink activity), as can be expected from the metabolic effector. However, a local effect of both factors on the delay of leaf senescence cannot be ruled out as CKs and cwInv are involved in the same signalling pathway regulating leaf senescence (Lara *et al.*, 2004), and the *CIN1* gene is expressed in leaves and other organs despite being driven by a putative fruit-specific promoter (Albacete *et al*., unpublished data). In this regard, the higher number of fruit in salinized transgenic plants can be explained by a lower flower abortion rate owing to less altered source–sink relationships (Ghanem *et al.*, 2009).

Finally, it can be stated that the interaction between hormones and sucrose metabolism is a key factor in the physiological effort of the plant to maintain tomato productivity under saline conditions, suggesting that the regulation of the source–sink relationships provides a good biotechnological approach to improve salt tolerance in horticultural crops (Albacete *et al.*, 2014). However, even if it can be assumed that the adaptation to stress has a cost in terms of productivity, much work will be needed to fine tune the individual hormonal and metabolic regulation pathways and their mutual interactions to optimize energetic status of the plant and maintain crop productivity under harmful conditions.
